# Increased hemoglobin and heme in MALDI-TOF MS analysis induce ferroptosis and promote degeneration of herniated human nucleus pulposus

**DOI:** 10.1186/s10020-021-00368-2

**Published:** 2021-09-08

**Authors:** Liang Shan, Ximing Xu, Jing Zhang, Peng Cai, Han Gao, Yingjie Lu, Jiangang Shi, Yinlong Guo, Yue Su

**Affiliations:** 1 Institute of Interdisciplinary Integrative Medicine Research, Shanghai University of Traditional Chinese Medicine, 1200 Cailun Road, Shanghai, 201203 People’s Republic of China; 2 National Center for Organic Mass Spectrometry in Shanghai, Shanghai Institute of Organic Chemistry, Chinese Academy of Sciences, 345 Lingling Road, Shanghai, 200032 People’s Republic of China; 3 Department of Orthopedics, Spine Surgery Section, Changzheng Hospital, Second Military Medical University, 415 Fengyang Road, Shanghai, 200003 People’s Republic of China; 4 Shanghai Institute of Nutrition and Health, Chinese Academy of Sciences, Shanghai, 200032 People’s Republic of China; 5 Department of Encephalopathy, Shanghai Municipal Hospital of Traditional Chinese Medicine, Shanghai University of Traditional Chinese Medicine, Shanghai, 200071 People’s Republic of China

**Keywords:** Lumbar disc herniation, Disc degeneration, Heme iron, Ferroptosis, Vasculogenesis, MALDI-TOF MS

## Abstract

**Background:**

Neovasculogenesis is characteristic of herniated lumbar discs, in which extruded nucleus pulposus is prone to heme iron-induced cytotoxicity (increased oxidative stress causing ferroptosis). However, recent analyses of neovascularization are very complicated, and the mechanism of action is rarely reported.

**Methods:**

Matrix-assisted laser desorption/ionization–time-of-flight mass spectrometry (MALDI-TOF MS) was performed to analyze human herniated and nonherniated nucleus pulposus. Then, the clinical relevance of the MALDI-TOF MS results and Pfirrmann classification of the degenerative nucleus pulposus were analyzed. To explore the mechanism, the heme-induced ferroptosis effect was evaluated at both the tissue and cell levels using high-resolution MALDI-TOF MS and molecular biology methods.

**Results:**

The spectra revealed that hemoglobin (Hb) and heme signals were greatly increased, thus serving as predictors of vasculogenesis in herniated nucleus pulposus. The clinical relevance analysis demonstrated that the intensity of Hb and heme peaks was closely related to the Pfirrmann classification of degenerative nucleus pulposus. Mechanistically, increased heme catabolism and downregulation of glutathione peroxidase 4 (GPX4) levels were detected in herniated nucleus pulposus, reflecting iron-dependent cell death or ferroptosis. Iron levels was also increased in herniated nucleus pulposus compared with that in nonherniated nucleus pulposus. Furthermore, accuracy mass measurements confirmed that the levels of ferroptosis-related metabolites, such as glutathione, arachidonic acid (AA), sphinganine, polyunsaturated fatty acid (PUFA), and tricarboxylic acid (TCA) cycle metabolites, were significantly different between herniated and nonherniated tissues, indicating that the interior of the herniated tissues is a pro-oxidant environment. Moreover, heme-induced ferroptosis was verified in human nucleus pulposus cells (HNPCs), and the underlying mechanism might be associated with the Notch pathway.

**Conclusions:**

Neovascularization in herniated nucleus pulposus may expose tissues to high levels of heme, which can induce cytotoxicity and ferroptosis within tissues and accelerate the progressive degeneration of herniated nucleus pulposus. This study is beneficial for understanding the pathological mechanism of herniated nucleus pulposus and facilitating the development of nonoperative interventions for treating lumbar disc herniation (LDH).

**Supplementary Information:**

The online version contains supplementary material available at 10.1186/s10020-021-00368-2.

## Background

Lumbar disc herniation (LDH) is a common cause of chronic low back pain, with a lifetime prevalence of 84%, and 11% of patients with LDH suffer serious disability (Yao et al. 2020). Disc degeneration is a primary reason for LDH, which is influenced by many factors, including age, histological structure, biomechanics, genes, inflammation, and osteoporosis (Qiu et al. 2020; Ala-Kokko 2002; Cazzanelli and Wuertz-Kozak 2020). In 1993, neovascularization was first observed by histological staining in herniated nucleus pulposus of LDH patients, and this phenomenon was found to be closely related to age and disease duration (Yasuma et al. 1976). Vasculogenesis may be a repair process after disc injury and plays a role in promoting tissue degradation (Xiao et al. 2020). Dale E. Fournier reported that discs should not be generalized as avascular tissues, and vascularization of discs differs based on the constituent tissues, age, and state of the degeneration or damage (Fournier et al. 2020). However, the mechanism of vasculogenesis in disc degeneration and LDH remains unclear.

Previous studies demonstrated that hemoglobin (Hb) is liberated from extravasated red blood cells (RBCs) in immature microvessels of atherosclerotic lesions and human neoplasms (Nagy et al. 2010; Jeney et al. 2014; Cermak et al. 1993). Then, the oxidation of Hb to ferryl hemoglobin (ferryl Hb) results in the release of heme moieties (ferriporphyrin), a major source of intracellular iron (Chifman et al. 2014). Additional file 1: Fig. S1 shows the degeneration process of heme catalyzed by heme oxygenase-1 (HO-1). Heme iron constitutes the prosthetic group for proteins that influences many fundamental biological processes, including catalyzing free radical reactions within cells, signal transduction, respiration, and energetic homeostasis (Chifman et al. 2014; Jeney et al. 2002; Hower et al. 2009; Yin et al. 2007). The aberrant accumulation of bioiron leads to nonapoptotic cell death caused by iron-dependent oxidative damage, namely, ferroptosis, which is mainly characterized by reduced cell volume and increased mitochondrial membrane density (Dixon et al. 2012). Heme-induced ferroptosis is related to many acute traumas and chronic degenerative lesions, such as atherosclerotic processes (Nagy et al. 2010), malignant tumors (Buss et al. 2004), and neurodegenerative diseases (Zhang et al. 2020a).

A histological feature of impaired discs is the formation of vascularized granulation tissues from the nucleus pulposus to the annulus fibrosus along pathological tears in the extruded tissues (Arai et al. 2000). Vascularized granulation tissues produce inflammatory cytokines, such as prostaglandin E2, interleukin-6, and interleukin-8, which stimulate nociceptors in the extruded nucleus pulposus and cause exacerbated clinical syndromes (Yasuma et al. 1976). RBC extravasation in disc neovascularization and heme iron-mediated cytotoxicity in herniated discs has not been reported in previous studies. Since immature vessels are leaky and prone to rupture, leading to the extravasation of RBCs, hemorrhage is features in atherosclerotic lesion (Nagy et al. 2010; Jeney et al. 2014) and human neoplasms (Cermak et al. 1993), which will cause iron to deposit in tissues. We speculated that immature vessels in herniated discs might also cause extravasation of RBCs and the deposition of iron in the tissues. Ferroportin (FPN) is the only known mammalian iron exporter, and it plays a pivotal role in regulating the cycling of iron in red blood cells. A recent study showed that dysfunctional FPN induced iron overload and ferroptosis in nucleus pulposus cells (Lu et al. 2021). Thus, neovasculogenesis in herniated nucleus pulposus may also be implicated in many pathological changes in LDH. However, recent studies on neovasculogenesis in herniated nucleus pulposus are lacking. In contrast to tumor tissues with abundant vessels, it is difficult to identify the vascularized zone during sectioning of newly vascularized granulation tissues in herniated nucleus pulposus, which greatly affects the reliability of the results. Additionally, in the progression of disc degeneration, a decrease in nucleus pulposus water was found to be concomitant with the degeneration of proteoglycan and collagen, causing nucleus pulposus to shrink (Arai et al. 2000). Therefore, it is a challenge to obtain structured histology sections in practice.

Matrix-assisted laser desorption/ionization time-of-flight mass spectrometry (MALDI-TOF MS) has advantages that include lower cost and automation and its use has led to considerable advances in the research of cancer, cardiovascular diseases, and infectious diseases (Wang et al. 2011; Wieczorek et al. 2020; Smaalen et al. 2019). The direct analysis of body fluids or tissue samples using MALDI-TOF MS can be performed to identify specific predictors and reveal characteristic pathological changes caused by diseases (Grant and Hoofnagle 2014).

In this study, MALDI-TOF MS was used to directly analyze herniated nucleus pulposus and nonherniated control nucleus pulposus with the aim of identifying differentially expressed molecules that can rapidly and accurately indicate pathologic features. In the practice of clinical diagnosis, the analysis of large proteins is much more complicated than that of small-molecule compounds (Chen et al. 2013). In this study, a commercial high-mass detector was incorporated with the MALDI-TOF MS system to detect intact highly abundant proteins in human nucleus pulposus. Although it has the advantages of celerity and accuracy, the cost is quite high. Accounting for this, the detection range of MS was also extended to the low-mass range (100–2000 Da) to screen small predictors, making the application more suitable for clinical and experimental applications. Furthermore, a heme-induced ferroptosis mechanism was first proven to be involved in the degeneration of herniated nucleus pulposus at both the tissue and cell levels.

## Methods

### Patients and tissue samples

In the herniated group, nucleus pulposus tissues of 16 patients with LDH (at L2/3 in one patient, at L3/4 in 5 patients, at L4/5 in 9 patients, and at L5/S1 in one patient) were obtained using a standard posterior microdiscectomy approach.

(1) Inclusion criteria: Complete preoperative radiologic images (X-ray, CT, and MRI) were available; all the herniated nucleus pulposus samples were obtained using a standard posterior microdiscectomy approach; the sample were classified as Pfirrmann grade 3 and higher. (2) Exclusion criteria: patients had lumbar fractures, inflammatory disease, lumbar spine tumor, spinal deformity, severe osteoporosis, or herniated scoliosis; patients have a history of lumbar spine surgery.

Nonherniated nucleus pulposus was obtained from 9 patients with tethered cord syndrome (TCS) (at L1/2 in one patient, at L2/3 in 2 patients, at L3/4 in 3 patients, and at L4/5 in 3 patients) using homogeneous spinal-shortening axial decompression. All patients provided informed consent before enrollment. The study was conducted in accordance with the Declaration of Helsinki, and the protocol was approved by the Medical Ethics Committee of Shanghai Changzheng Hospital. The clinicopathological characteristics of the patients are shown in Table 1.

**Table 1:** Clinicopathological characteristics of LDH patients and control patients with TCS

Patients ID	Samples	Gender	Ages	Pfirrmann	Segment
1	TCS	Female	18	1	L4/L5
2	TCS	Male	18	1	L4/L5
3	TCS	Male	28	1/2	L3/L4
4	TCS	Female	36	1/2	L4/L5
5	TCS	Female	18	1	L1/L2
6	TCS	Female	18	1	L2/L3
7	TCS	Female	18	1	L3/L4
8	TCS	Male	19	1	L2/L3
9	TCS	Male	19	1	L3/L4
10	LDH	Female	51	4	L3/L4
11	LDH	Male	40	3	L4/L5
12	LDH	Male	22	3/4	L4/L5
13	LDH	Female	28	4	L4/L5
14	LDH	Male	62	4	L4/L5
15	LDH	Male	63	4	L3/L4
16	LDH	Male	63	3	L3/L4
17	LDH	Female	67	3/4	L5/S1
18	LDH	Male	37	3	L3/L4
19	LDH	Male	64	4/5	L3/L4
20	LDH	Male	56	4/5	L4/L5
21	LDH	Male	55	4	L4/L5
22	LDH	Female	67	4	L4/L5
23	LDH	Female	47	4	L4/L5
24	LDH	Female	54	4/5	L2/L3
25	LDH	Female	54	3	L4/L5

### Cell culture

Human primary nucleus pulposus cells (HNPCs) were provided by Shanghai YCBIO Co., Ltd. Cells were cultured in DMEM with 10% fetal bovine serum and 1% penicillin–streptomycin in a 37 °C cell culture incubator with 5% CO_2_. Hb (Sigma, H7379), heme (Sigma, 51,281), deferoxamine mesylate salt (DFO) (Shycbio, Y1310), and ferric ammonium citrate (FAC) (Sigma, F5879) were dissolved in DMEM to a final concentration of 20 mg/mL. The ferroptosis inducer erastin (Sigma, E7781) was dissolved in DMSO to a final concentration of 1 mg/mL. The samples were stored at 4 ℃ for short-term use.

### Protein extraction

After thawing, the tissues were washed twice with deionized water to remove blood contamination on the surface. Then, samples were minced with scissors on ice and homogenized in RIPA buffer (Solarbio, R0010) containing 1% phenylmethylsulfonyl fluoride (PMSF) at a concentration of 10 μL/mg. Then, the homogenates were lysed at 4 ℃ for 30 min and centrifuged at 12,000*g* for 30 min at 4 ℃. For cell protein extraction, the cells were lysed using 100 μL of RIPA buffer (Solarbio, R0010) containing 1% PMSF after treatment for 24 h in 6-well plates and then centrifuged at 12,000*g* for 30 min at 4 ℃. Supernatants were collected as protein samples and stored at −80 ℃ until use.

### MALDI-TOF MS analysis

The matrix was dissolved in a 1:1 mixture of acetonitrile and water (0.1% trifluoroacetic acid) to a final concentration of 10 mg/mL. Sinapic acid was used as the matrix for the Hb analysis, and dihydroxybenzoic acid was used as the matrix for the heme analysis. Before the MALDI-TOF MS analysis, 1.5 μL of a mixture containing protein samples and matrix solution (1:40) was added dropwise onto a MALDI plate and dried at room temperature. For the Hb analysis, the spectra were collected by MALDI-TOF MS using a Shimadzu Biotech LaunchPad (Shimadzu, Japan) equipped with a high-mass detector (CovalX AG, Switzerland) in Linear-CovalX mode. For the heme analysis, the spectra were collected by MALDI-TOF MS using a Shimadzu Biotech LaunchPad (Shimadzu, Japan) in reflectron mode. Ionization was achieved using a N2 laser (337 nm) and 100 laser shots for each mass spectrum. The spectra were calibrated using external standards. The accuracy of the mass of the metabolites (*m/z* = 50–1000) was analyzed by MALDI Spiral TOF-TOF MS (JEOL, Japan) in spiral mode. Ionization was achieved using a Nd:YLF laser (349 nm) with 2000 laser shots for each mass spectrum. Then, the Human Metabolome Database (HMDB) (http://www.hmdb.ca/) was used to identify potential predictors.

### Hematoxylin and eosin (H&E) staining

Nucleus pulposus was embedded in paraffin blocks and then cut into 7 μm sections. Histological examinations were performed by H&E staining of the deparaffinized sections of these tissues.

### Atomic absorption spectrometry

Iron contents in tissues were determined by atomic absorption spectrometry (ZEEnit 770P, Germany).

### Prussian blue staining

Nucleus pulposus was embedded in paraffin blocks and then cut into 7 μm sections. The iron staining was performed using Tris-buffered saline containing 0.025% 3-diaminobenzidine tetrahydrochloride.

### Western blot analysis

Protein concentrations were determined with a BCA protein assay kit (Beyotime, P0012i). Twenty to thirty micrograms of total protein was separated by 10% SDS-PAGE (EpiZyme, pg112) and then transferred onto nitrocellulose membranes (Pall, 66485). Nonspecific sites were blocked with 5% nonfat milk in phosphate-buffered saline (with 0.1% Tween-20) at room temperature. Next, the blots were incubated overnight at 4 ℃ with the following primary antibodies: anti-Hbα (Santa Cruz, sc-514378), anti-Hbβ (Santa Cruz, sc-21757), anti-CD31 (Affinity, AF6191), anti-glutathione peroxidase 4 (GPX4) (Affinity, DF6701), anti-heme oxygenase-1 (HO-1) (Affinity, AF5393), anti-Bcl2 (ImmunoWay, YM3041), anti-Bax (ImmunoWay, YT0455), anti-Notch1 (Cell Signaling Technology, #3608), anti-Jag1 (Cell Signaling Technology, #70109), anti-Hes1 (Abcam, ab108937), anti-Hey1 (Abcam, ab154077), and anti-β-actin (Affinity, AF7018). The next day, the blots were incubated with horseradish peroxidase (HRP)-conjugated secondary antibodies (Affinity, s0001) and visualized with New Super ECL assay kit (KeyGen BioTECH, KGP1128).

### Cell viability analysis

A total of 10,000 cells were inoculated in 96-well plates and treated for 24 h. Then, the medium was replaced with 10 μl of CCK-8 (Dojindo, CK04) and 100 μl of DMEM, and then, the cells were incubated for 1 h at 37 ℃. The optical density (OD) at 450 nm was read using a microplate reader.

### Immunohistochemistry (IHC)

Nucleus pulposus tissues were embedded in paraffin blocks and then cut into 7 μm sections. For IHC, a one-step IHC assay (KeyGen, KGOS60) was used to stain 7 μm thick sections, and the paraffin sections were incubated with anti-GPX4 (Affinity, DF6701) and HO-1 (Affinity, AF5393).

### ROS levels

Total ROS levels in the HNPCs were analyzed using a reactive oxygen detection kit (Abcam, ab236206) according to the manufacturer’s protocol. The data are expressed as relative fluorescent units (RFUs) normalized to cell number.

### qRT- PCR

Total RNA was extracted using TRIzol Reagent, and cDNA was synthetized with a RevertAidTM First Strand cDNA Synthesis Kit (Fermentas, Vilnius). Then, qPCR was performed using a PCR amplification kit (TaKaRa, R011). The specific primers are listed in Table 2.

**Table 2:** Primers used for qRT-PCR

Name	Sequences (5’–3’)
GAPDH-F	AACAGCCTCAAGATCATC
GAPDH-R	ACTGTGCAACCGTCACCC
Notch1-F	CAAAGTGTCTGAGGCCAG
Notch1-R	GTGAGTAAGACCAACAGC
Notch2-F	CTGCATGAACCATGGTCT
Notch2-R	ACCGTTCCTAACCGTTCC
Jag1-F	CAAGTGCACCCGCGACGA
Jag1-R	CCGTCGTGCTACGCCAAC
Jag2-F	CGCTGCGGAACGTGAACG
Jag2-R	GGAACCGGACCATGAGGA
Hes1-F	CGGCTGCGCTGAGCACAG
Hes1-R	CGCGCTTGCCGCGCACGA
Hes2-F	CTGCCTGGTCACTGCTCT
Hes2-R	CGGATCCTCACCTCCACT
Hey1-F	AGCAAGGATCTGCTAAGC
Hey1-R	GCATCAACAACTCTACGC

### Statistical analysis

Comparisons between two populations were performed using two independent sample t-tests. Differences between the treatment group and the control group were analyzed by Student's t-test. Correlations were assessed by Pearson correlation. Principal component analysis (PCA) and receiver operating characteristic (ROC) curve analyses were performed using the MALDI-TOF MS data with SPSS 21.0 (SPSS Inc., USA). A P value less than 0.05 was considered statistically significant.

## Results

### Hb signals were highly increased in herniated nucleus pulposus compared to nonherniated nucleus pulposus

**Fig. 1:**
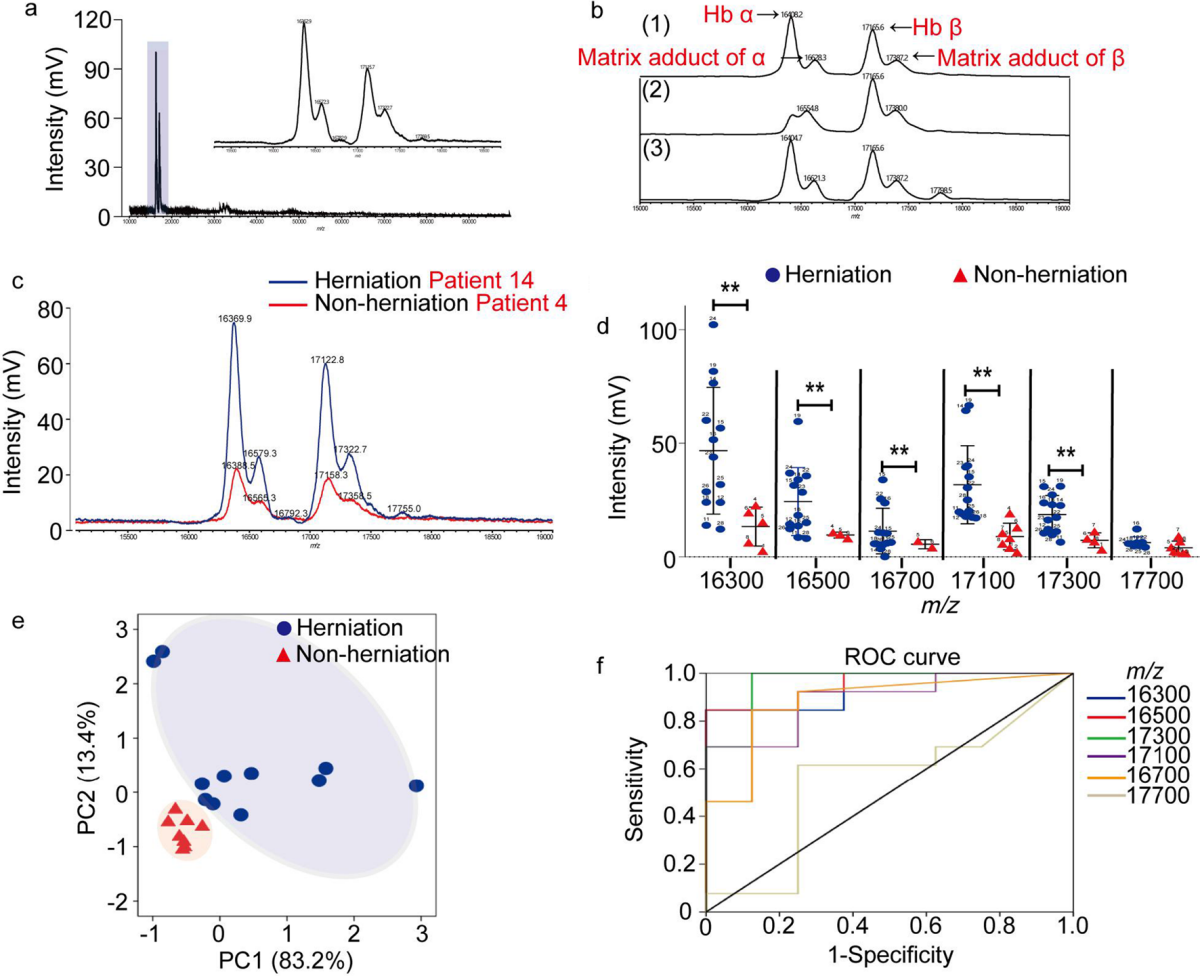
The levels of Hb in herniated nucleus pulposus are higher than those in nonherniated nucleus pulposus, as determined by MALDI-TOF MS. **a** MS spectra of total protein lysates measured by a high-mass detector. The tissue sample was obtained from the patient 24 in Table 1. **b** The spectra of Hb standard (1), tissue lysates (2), and a standard and lysate mixture (3) measured by the high-mass detector. The tissue sample was obtained from the patient 23 in Table 1. **c** The spectra of tissue lysates from herniated nucleus pulposus and nonherniated nucleus pulposus measured by a high-mass detector. The tissue samples were obtained from the patients 14 and 4 in Table 1. **d** Scatter plot of the Hb-related peak intensity in 13 samples of herniated nucleus pulposus and 9 samples of nonherniated nucleus pulposus as measured by a high-mass detector. The data were analyzed with GraphPad Prism 5.02 software. **P < 0.01. **e** The PCA clustering diagram of 13 samples of herniated nucleus pulposus and 9 samples of nonherniated nucleus pulposus. **f** ROC curve analysis of 6 Hb-related peaks in 13 samples of herniated nucleus pulposus and 9 samples of nonherniated nucleus pulposus

First, tissue lysates from herniated nucleus pulposus were analyzed by MALDI-TOF MS combined with a high-mass detector. As shown in Fig. 1a, some high-intensity peaks were observed in the high-mass range (10–100 kDa). Through further analysis of the spectra of Hb standards, tissue lysates, and a mixture of standards and lysates, these peaks were finally identified as Hb-related peaks. The protein peaks with *m/z* values of 16300 and 17100 were ascribed to Hb α and Hb β, respectively, which are subunits of Hb (Fig. 1b). Then, we observed that Hb-related peaks clearly illustrated different intensities in herniated and nonherniated nucleus pulposus (Fig. 1c).

The variability of Hb contents in different tissues is one of the indicators for assessing vasculogenesis. For this reason, a high level of Hb may be important in the diagnosis of vasculogenesis in herniated nucleus pulposus. To verify this supposition, 13 samples of herniated nucleus pulposus and 9 samples of nonherniated nucleus pulposus were analyzed by MALDI-TOF MS with a high-mass detector. Figure 1d shows that Hb-related peaks with *m/z* values of 16300, 16500, 16700, 17100, and 17300 were differentially upregulated in herniated nucleus pulposus compared with nonherniated nucleus pulposus (P < 0.01), except for the protein peak with an *m/z* value of 17700, suggesting that high levels of Hb are a specific pattern in herniated nucleus pulposus. Next, the high-resolution mass spectrometry (MS) data were processed using PCA. The first (83.2%) and the second (13.4%) principal components were chosen for visualization. As shown in Fig. 1e, clear separation of herniated nucleus pulposus and nonherniated control nucleus pulposus was obtained. The data were classified into normal and diseased groups and they exhibited variations, indicating heterogeneity between these two groups. To further explore the diagnostic value of various Hb-related peaks in herniated nucleus pulposus, we used the ROC curve to analyze the MS data. As expected, the ROC analysis revealed the ability of the 5 protein peaks to predict vasculogenesis positively in herniated nucleus pulposus, suggesting that Hb might be a candidate predictor for vasculogenesis in herniated nucleus pulposus (Fig. 1f).

### Heme contents were increased specifically in herniated nucleus pulposus

**Fig. 2:**
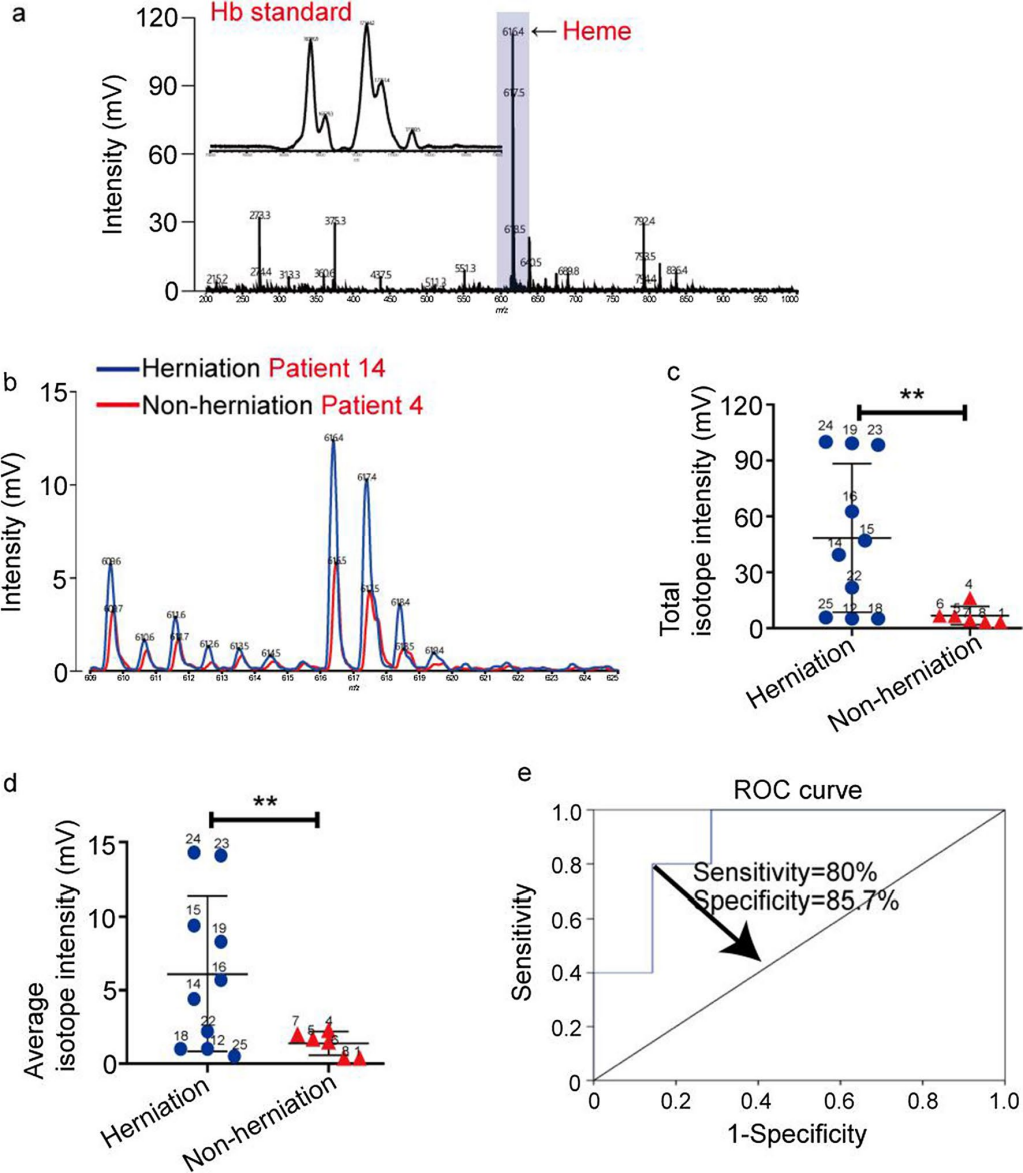
Specific increase in heme content in herniated nucleus pulposus as analyzed by MALDI-TOF MS. **a** MS spectra of Hb standard at a low-mass range. **b** The spectra of tissue lysates from herniated nucleus pulposus and nonherniated nucleus pulposus in the low-mass range. The tissue samples were obtained from the patients 14 and 4 in Table 1. **c**–**d** The total intensity (**c**) and average intensity (**d**) of heme and its isotope peaks in 10 samples of herniated nucleus pulposus and 6 samples of nonherniated nucleus pulposus. The data were analyzed with GraphPad Prism 5.02 software. **P < 0.01. **e** ROC curve analysis of the heme peaks of 10 samples of herniated nucleus pulposus and 6 samples of nonherniated nucleus pulposus

Next, the detection range of MS was extended to the low-mass range to screen small-molecule predictors that may be the most suitable for clinical and experimental applications. Heme is the prosthetic group of Hb, and the result from Fig. 2a showed heme peak (*m/z* = 616.4) in Hb standard. Figure 2b indicates that the heme and its isotopes had elevated levels in herniated nucleus pulposus compared to the normal control, suggesting that the content of heme in the nucleus pulposus directly reflects Hb levels (Fig. 2a). Then, the intensity of heme-related peaks in 10 samples of herniated nucleus pulposus and 6 samples of nonherniated nucleus pulposus was detected. The scatter plots revealed significant differences in the total intensity (Fig. 2c) and average intensity (Fig. 2d) between these two groups. The heme was specifically increased in the herniated nucleus pulposus, which was consistent with the Hb analysis results and revealed pathological features of vasculogenesis in the herniated nucleus pulposus. Moreover, ROC curves were generated to assess the diagnostic value of heme in herniated nucleus pulposus. In this model, all data showed good diagnostic accuracy, and one data point predicted vasculogenesis with a sensitivity of 80% and specificity of 85.7% (Fig. 2e).

### Hb and heme levels are positively correlated with the Pfirrmann classification of disc degeneration

**Fig. 3:**
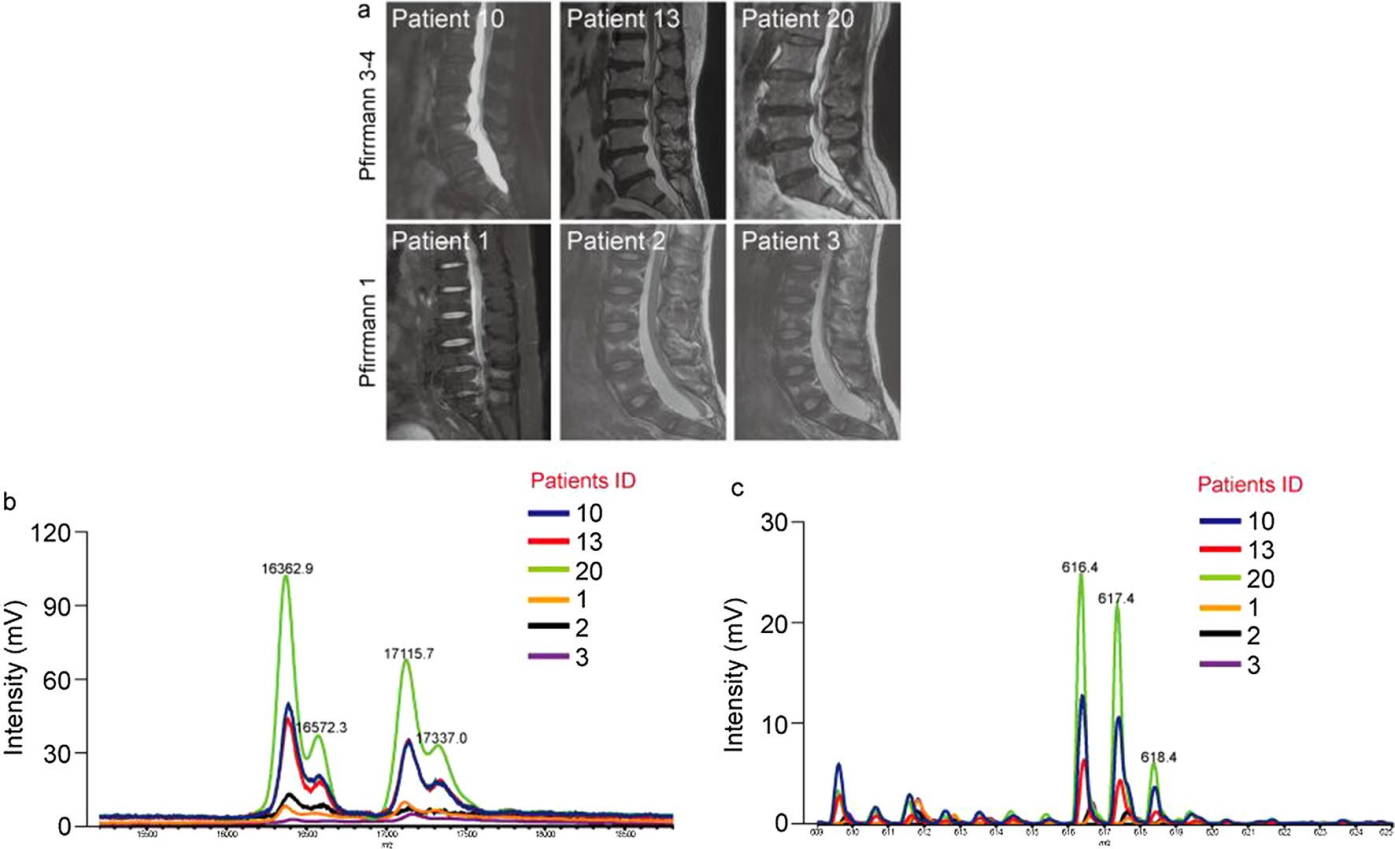
Hb and heme levels are positively correlated with the Pfirrmann classification of disc degeneration. **a** Representative MRI images from 3 patients with LDH and 3 control patients with tethered cord syndrome. **b** The spectra corresponding to the MRI images measured by a high-mass detector. **c** The spectra corresponding to MRI images in the low-mass range

Clinically, disc degeneration is graded according to the alteration in T2WI signal in the parasagittal MRI images of dysfunctional segments combined with Pfirrmann classification. As shown in Fig. 3a, the nucleus pulposus with Pfirrmann grade 1 was uniform and well demarcated with the annulus, while the nucleus and annulus in the sample with Pfirrmann grade 3–4 had poorly defined margins. The signal of the herniated nucleus pulposus with Pfirrmann grade 3 or 4 was reduced and was gray-black in the MRI images. Next, we verified the correlation between vasculogenesis and the clinicopathology of herniated nucleus pulposus. First, representative H&E staining showed that the chondrocytes and nuclear cell structures were clearly present in the nucleus pulposus with Pfirrmann grade 1, and the surrounding collagen had formed a regular network and showed had no significant degeneration. The number of cells in the herniated nucleus pulposus with Pfirrmann grade 3–4 was markedly reduced. In addition, severe degenerative changes, such as neovasculogenesis, were clearly observed in the cell matrix (Additional file 1: Fig. S2). The MS signal intensity of Hb and heme in the surgically removed herniated nucleus pulposus was compared with the MRI images of the herniated nucleus pulposus taken before surgery. The intensity of the Hb-related peaks in the herniated nucleus pulposus with Pfirrmann grade 3–4 was significantly higher than that in the nucleus pulposus with Pfirrmann grade 1 (Fig. 3b). Hb was increased as a result of vasculogenesis; therefore, vasculogenesis might be closely associated with LDH progression. Furthermore, a similar result was observed with the heme analyzed by MALDI-TOF MS. The results shown in Fig. 3c reveal that the peak intensity of heme in the herniated nucleus pulposus with Pfirrmann grade 3–4 was significantly higher than that in the nucleus pulposus with Pfirrmann grade 1.

**Fig. 4:**
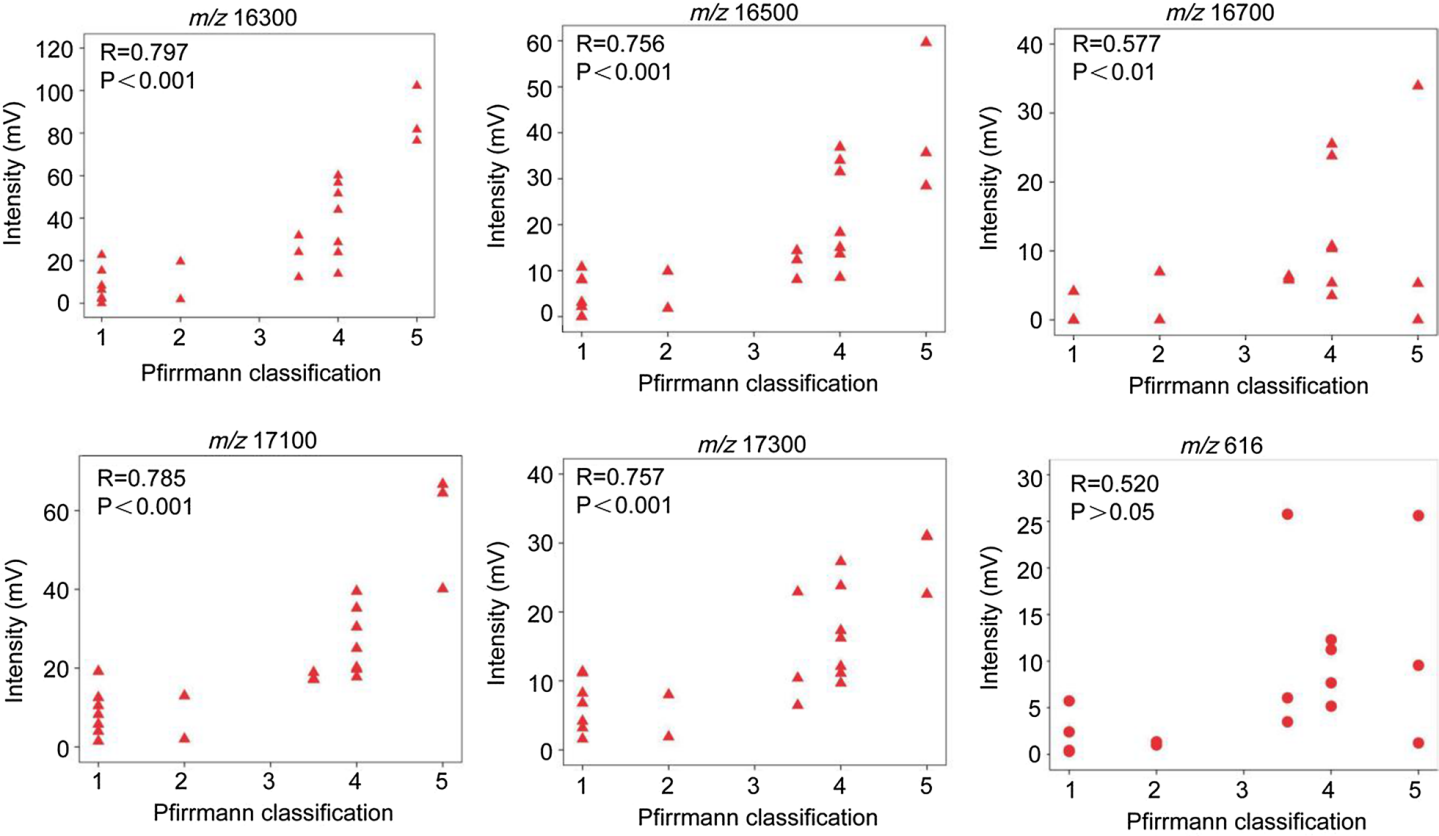
Pearson correlation analysis of Pfirrmann classification and intensity of MALDI-TOF MS peaks

Furthermore, a correlation analysis was performed between the results of MALDI-TOF MS and the Pfirrmann classification of disc degeneration. As shown in Fig. 4, there was a significant linear correlation between the intensity of Hb-related peaks and the Pfirrmann classification. A strong correlation was defined as an R ratio between 0.6 and 0.8 (*m/z* = 16300, 16500, 17100, and 17300), a moderate correlation was an R ratio between 0.4 and 0.6 (*m/z* = 16700), and a weak correlation as an R ratio between 0.2 and 0.4 (*m/z* = 616).

### High levels of Hb and heme induce ferroptosis in herniated tissues

**Fig. 5:**
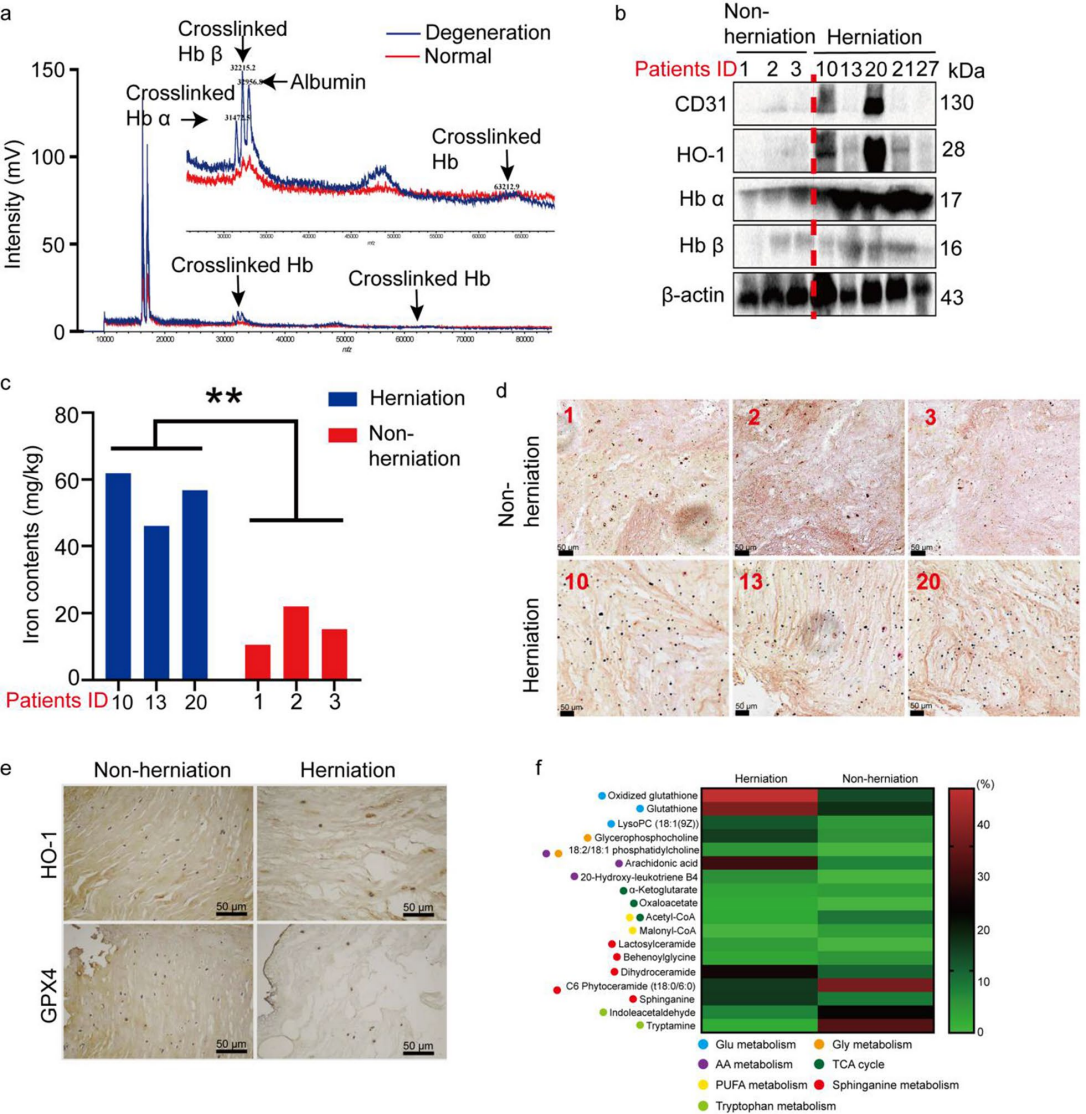
High levels of Hb and heme induce ferroptosis in herniated tissues. **a** Hb polymers in herniated nucleus pulposus were detected by MALDI-TOF MS. **b** The protein levels of CD31, HO-1, Hb α, and Hb β in 5 samples of herniated nucleus pulposus (Pfirrmann scores of 3–4) and 3 samples of nonherniated nucleus pulposus. **c** Iron levels in 3 samples of herniated nucleus pulposus (Pfirrmann scores of 3–4) and 3 samples of nonherniated nucleus pulposus. **d** Prussian blue staining of herniated nucleus pulposus (Pfirrmann grade 3–4) and nonherniated nucleus pulposus. **e** HO-1 and GPX4 protein levels in herniated nucleus pulposus (Pfirrmann grade 3–4) and nonherniated nucleus pulposus, as determined by immunohistochemical analysis. Representative IHC images were taken at a magnification of 200× . **f** Identification of ROS and ferroptosis-related metabolites in herniated nucleus pulposus (Pfirrmann grade 3–4) and nonherniated nucleus pulposus by high-resolution MALDI-TOF MS

The invaded blood vessels in the nucleus pulposus may not be critical for the initiation of disc degeneration or LDH, but secondary vasculogenesis can promote degeneration progression. Interestingly, the MALDI-TOF MS results indicated that the increase in Hb was accompanied by the formation of crosslinked Hb, a predictor of the preceding formation of ferryl Hb, implying that Hb was oxidized and heme iron dissociated from the resultant ferryl Hb (Fig. 5a) (Posta et al. 2020). It is tempting to speculate that the released iron induce further oxidative stress and ferroptosis in herniated nucleus pulposus. To test this hypothesis, WB was performed to analyze the levels of Hbα and Hbβ, the endothelial markers CD31 and HO-1, and the ferroptosis suppressor GPX4 in 5 samples of herniated nucleus pulposus and 3 samples of nonherniated nucleus pulposus. As shown in Fig. 5b, consistent with the MALDI-TOF MS results, herniated tissues showed increased Hbα and Hbβ protein levels compared with the normal group. CD31 was significantly overexpressed in two samples of herniated tissues, while it was negligibly expressed in the control group, which verified the reliability of the aforementioned results based on MALDI-TOF MS. Furthermore, HO-1, generated in response to heme catabolism, was highly expressed in the two degraded tissues with high CD31 levels, and it can induce reactive oxygen species (ROS) production and ferroptosis in herniated nucleus pulposus (Fig. 5b). Next, an atomic absorption spectrophotometer was performed to further confirmed the presence of iron in 3 samples of herniated nucleus pulposus (Pfirrmann scores of 3–4) and 3 samples of nonherniated nucleus pulposus. The results showed that the iron contents in herniated nucleus pulposus were significantly higher than that in nonherniated nucleus pulposus (Fig. 5c). As shown in Fig. 5d, Prussian blue staining indicated herniated nucleus pulposus contained more dark iron granules compared with nonherniated nucleus pulposus, which was consistent with the results of tissue iron content determination (Fig. 5d). The above results also confirmed the RBC extravasation and hemolysis in neovessels of herniated nucleus pulposus.

Moreover, the IHC results showed that although the number of cells was significantly decreased and histological structures were lost, HO-1 proteins were specifically expressed in herniated nucleus pulposus compared to nonherniated nucleus pulposus (Fig. 5e).

Finally, ROS-related metabolites in herniated and nonherniated nucleus pulposus were identified by high-resolution MALDI-TOF MS. As shown in Fig. 5f, in the nonherniated nucleus pulposus, the number and intensity of detectable metabolites were significantly reduced compared to those in the herniated nucleus pulposus. Through accuracy mass measurements, a total of 28 endogenous metabolites were identified in the herniated nucleus pulposus, and all these metabolites are associated with ferroptosis-related metabolic pathways involved in glutathione metabolism, glycine metabolism, arachidonic acid (AA) metabolism, sphinganine metabolism, polyunsaturated fatty acid (PUFA) metabolism, and the tricarboxylic acid (TCA) cycle. More detailed information of metabolites were summarized in Additional file 2: Table S1 and the MS spectra were shown in Additional file 1: Fig. S3. These results suggested a state of high oxidative stress in the interior of the herniated nucleus pulposus, which likely accelerates disc degeneration.

### Heme induces ferroptosis of human nucleus pulposus cells

**Fig. 6:**
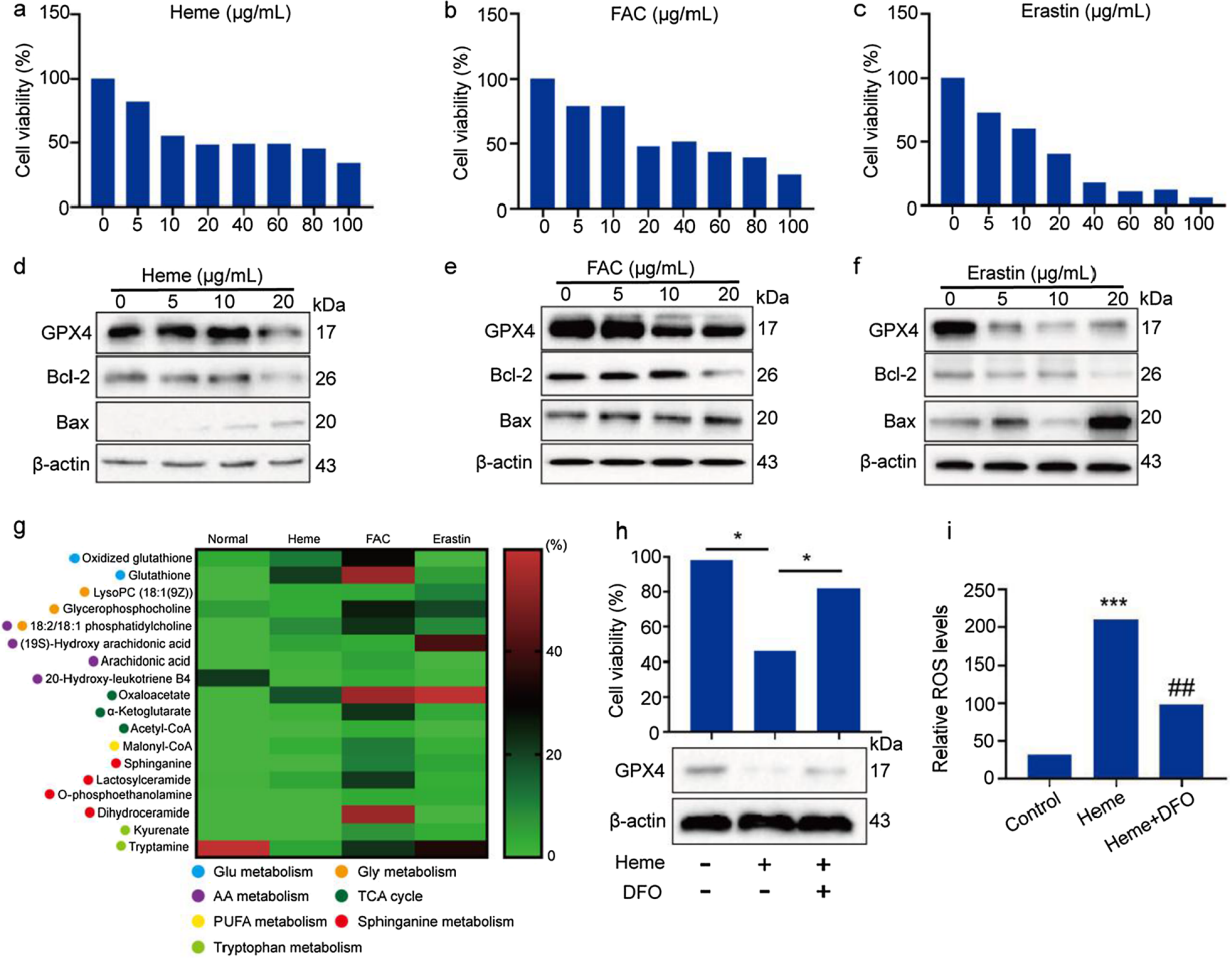
Heme induces ferroptosis in human nucleus pulposus cells. **a**–**c** Viability of HNPCs was measured by CCK-8 after treatment with heme (**a**), FAC (**b**), and erastin (**c**) at concentrations of 0, 5, 10, 20, 40, 60, 80, and 100 μg/mL for 24 h. The data were analyzed with GraphPad Prism 5.02 software. **d**–**f** The protein levels of GPX4, Bcl-2, and Bax in HNPCs treated with heme (**d**), FAC (**e**), and erastin (**f**) at concentrations of 0, 5, 10, and 20 μg/mL for 24 h. **g** Viability of HNPCs was measured by CCK-8 after 20 μg/mL heme treatment for 24 h with or without 30 μg/mL DFO pretreatment for 1 h (upper), and the protein levels of GPX4 in HNPCs were measured after 20 μg/mL heme treatment for 24 h with or without 30 μg/mL DFO pretreatment for 1 h (lower). **h** Identification of ROS and ferroptosis-related metabolites in HNPCs after treatment with 20 μg/mL heme, 20 μg/mL FAC, and 10 μg/mL erastin for 24 h using high-resolution MALDI-TOF MS. **i** ROS levels in HNPCs after 20 μg/mL heme treatment for 24 h with or without 30 μg/mL DFO pretreatment for 1 h. The data were analyzed with GraphPad Prism 5.02 software. *P < 0.05, **P < 0.01

To confirm the effects of heme iron on the nucleus pulposus, HNPCs were treated with different concentrations of heme, and erastin and FAC were used as positive ferroptotic controls. For the setting of the heme concentration range, in pilot experiments performed previously, we compared the MALDI-TOF MS signal intensity of heme standard with the signal of the patients’ tissues, and roughly determined a range (data not shown). As shown in Fig. 6a–c, the inhibitory effects of heme, FAC, and erastin on cell viability were dose-dependent in the HNPCs. As shown in Fig. 6d–f, GPX4 protein levels were also decreased after treatment with heme, FAC, and erastin in the HNPCs, indicating that all three of these treatments can induce intracellular ROS production and cell death. The antiapoptotic molecule Bcl-2 and proapoptotic molecular Bax mark the occurrence of cell apoptosis. The results shown in Fig. 6d indicate that Bcl-2 protein levels were downregulated when HNPCs were treated with 20 μg/ml heme, whereas Bax levels were increased when HNPCs were treated with 10 μg/ml heme. Considering the results shown in Fig. 6a, we deduced that heme-induced ferroptosis occurred before apoptosis in these HNPCs. Furthermore, ROS-related metabolic pathways in the HNPCs were identified by high-resolution MALDI-TOF MS after heme, FAC, or erastin treatment (Fig. 6g). Similar to the analysis results at the tissue level, the number and intensity of detectable metabolites were significantly increased after heme and FAC treatment of HNPCs. These differentially expressed metabolites are involved in glutathione metabolism, glycine metabolism, AA metabolism, sphinganine metabolism, PUFA metabolism, and the TCA cycle. The number of detectable metabolites in the erastin group was also decreased compared to that in the heme and FAC groups. The reason is that the inhibitory effects of erastin on HNPCs were greater, and high death rates among cells weakened the MS signals (Fig. 6g). More detailed information on these metabolites is summarized in Additional file 2: Table S2 and the MS spectra are shown in Additional file 1: Fig. S4.

Although heme induced a decrease in cell viability, this negative outcome was significantly attenuated by cotreatment with DFO, demonstrating that heme-induced cell death was iron-dependent (Fig. 6h). Additionally, our results confirmed that heme-mediated inhibition of the GPX4 protein was rescued by DFO in HNPCs (Fig. 6h). To determine whether heme iron can disrupt the redox balance in HNPCs, total ROS levels were assessed. As shown in Fig. 6i, a significant upregulation of ROS levels was observed in HNPCs after heme treatment, while heme-induced ROS was inhibited by DFO. Collectively, these findings reaffirm the ferroptotic effects induced by heme iron in HNPCs.

### Heme-induced ferroptosis might be mediated by the Notch pathway in HNPCs

**Fig. 7:**
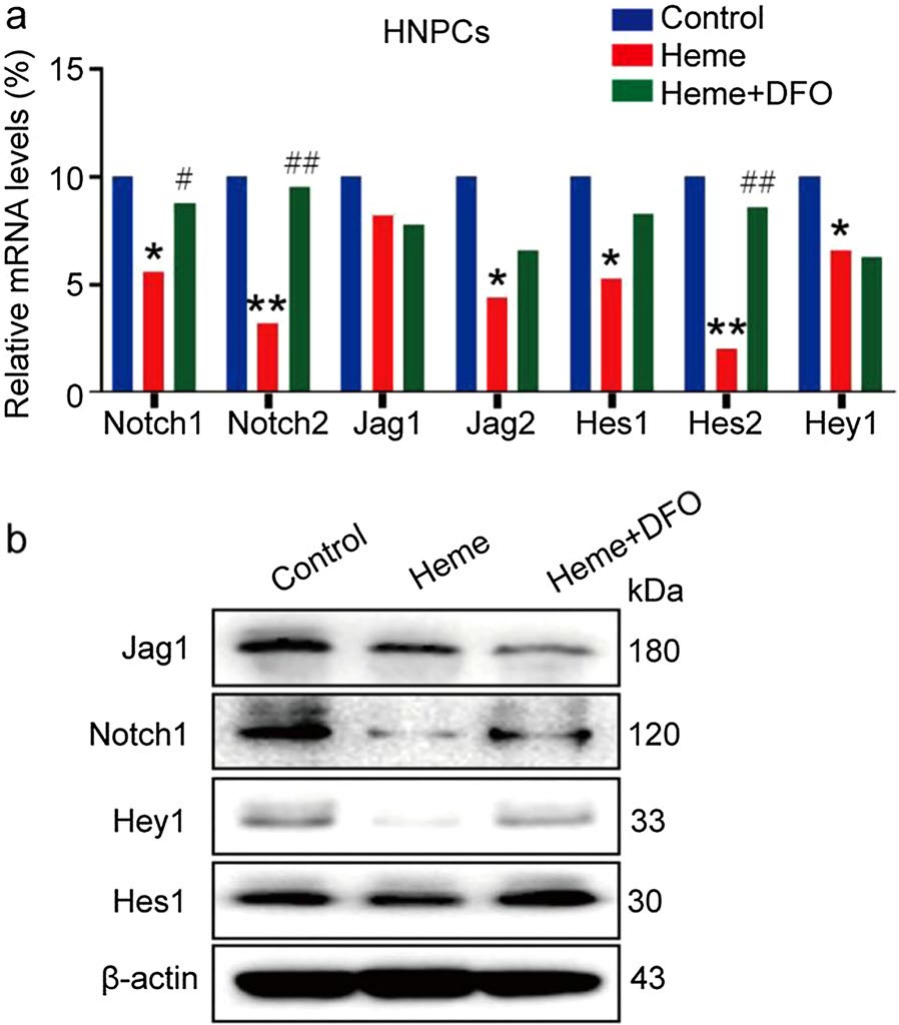
Changes in the Notch signaling pathway in HNPCs after heme treatment. **a** Notch-related mRNAs in HNPCs were measured by qRT-PCR after 20 μg/mL heme treatment for 24 h with or without 30 μg/mL DFO pretreatment for 1 h. Data were analyzed with GraphPad Prism 5.02 software. *P < 0.05, **P < 0.01. **b** Notch-related proteins in HNPCs were measured by western blot analysis after 20 μg/mL heme treatment for 24 h with or without 30 μg/mL DFO pretreatment for 1 h

Since numerous studies have reported that inhibition of the Notch signaling pathway is involved in disc degeneration (Xiong et al. 2020; Long et al. 2019), we investigated the relevant protein and mRNA changes in Notch signals after heme treatment. As depicted in Fig. 7a and b, there were significant changes in the protein and mRNA levels of Notch1, Notch2, Jag1, Jag2, Hes1, Hes2, and Hey1 after heme treatment of HNPCs. Notably, we also found that heme-induced inhibition of Notch-related molecules was effectively rescued by DFO cotreatment. These results suggest that nucleus pulposus degeneration induced by heme iron might be associated with the Notch signaling pathway.

## Discussion

MALDI-TOF MS can quickly monitor the *m/z* of molecules and provide mass information on samples. For this measurement, chemical reagents that react with specific molecules in samples are not needed, effectively avoiding false positive or false negative results (Smaalen et al. 2019; Grant and Hoofnagle 2014). In this study, MALDI-TOF MS was performed with a high-mass detector that did not induce saturated effects in the presence of a complex mixture over a broad mass range. As reported in our study, the increased Hb/heme levels in herniated nucleus pulposus are novel findings. Our research has some shortcomings, such as small sample sizes and age unmatched control groups. Disc degeneration is closely related to age and disease course. Subtle degeneration begins in humans at the age of 2 years, and the progressive herniation process with increasing age is the causeof LDH. Hence, most inpatient surgical populations with LDH are middle-aged and elderly people (Xiao et al. 2020). In contrast, TCS commonly affects children and young people. Hence, patients in the LDH group were older than the patients in the TCS group. Nevertheless, in order to exclude biases related to aging, future studies should examine age matched patients with various levels of disc degeneration to better support the present results. We believe that a reasonable clinical design will be implemented to verify the correlation of Hb/heme and the degeneration of herniated nucleus pulposus, which is likely to be clinically useful. In addition, our result was believed to be unaffected by blood contamination because all samples were washed before extraction and prepared with a standard assay. Moreover, Hb polymers were detected in herniated nucleus pulposus using a high-mass detector, marking the formation of ferryl Hb (oxidized Hb), which distinguishes heme iron from ferryl Hb. Then, the release of iron leads to further endogenous oxidative stress and ultimately to cytotoxicity (Sardar Pasha et al. 2021; Gbotosho et al. 2020). The results from MALDI-TOF MS demonstrated that both Hb and heme contents were positively correlated with the Pfirrmann classification of disc degeneration. Considering this, we proposed that heme-induced ferroptosis is involved in the process of disc degeneration.

Ferroptosis is mainly induced by the inactivation of the membrane lipid repair enzyme GPX4, which causes the accumulation of ROS among membrane lipids, a process that is iron dependent (Forcina and Dixon 2019). HO-1, a key enzyme for heme iron degradation, also plays the dual role of pro-oxidant and antioxidant. Studies have shown that HO-1 can not only enhance the chemotherapeutic sensitivity of cancer cells but can also induce cell death by regulating iron homeostasis (Waza et al. 2018). As expected, in this study, elevated levels of HO-1 were found while GPX4 levels were decreased in herniated tissues compared with nonherniated nucleus pulposus, indicating a state of high oxidative stress in the interior of herniated nucleus pulposus.

For high-resolution MALDI-TOF MS analysis of nucleus pulposus, the difference in MS signal intensity of oxidative markers between normal and herniated tissues might be a result of heme iron in the latter tissues. In addition to the heme iron we report, endogenous homocysteine and interleukin-1 can induce oxidative stress and degeneration of the nucleus pulposus (Zhang et al. 2020b,^ 2019^).

Erastin can inhibit the cystine-glutamate transporter in the membranes and reduce cystine uptake by cells, which hinders the synthesis of glutathione, the substrate of GPX4. Finally, ferroptosis is usually produced as a result of lipid ROS generation (Forcina and Dixon 2019). Here, for the first time, our results demonstrated that heme induced the same lethal effects on HNPCs as erastin and FAC. Significantly, heme-induced ferroptosis of HNPCs was inhibited by DFO, and these results might provide some ideas for the nonsurgical intervention in LDH. Perhaps clinically, it is possible to delay disc degeneration by inhibiting vasculogenesis or regulating iron metabolism in LDH patients.

**Fig. 8:**
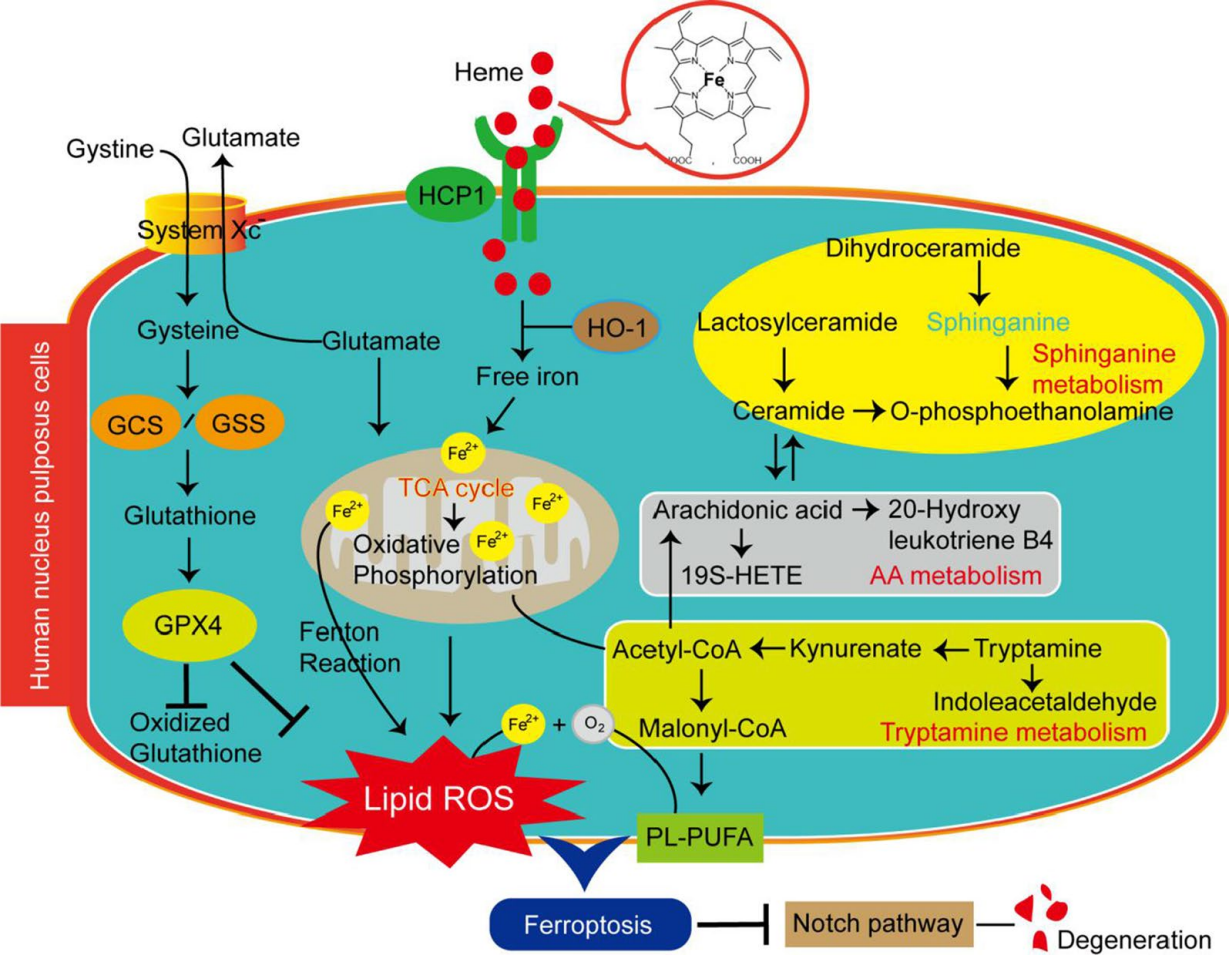
A potential mechanism by which heme iron induces ferroptosis and degeneration of human nucleus pulposus cells. Heme enters the cell through carrier proteins and is degraded by HO-1. This process releases free ferrous ions. Excessive free ferrous iron is oxidized to trivalent iron through the Fenton reaction in the presence of hydrogen peroxide that is metabolized by the cell, forming dangerous hydroxyl free radicals, which lead to lipid peroxidation and ferroptosis (middle). The substrates of peroxidation are polyunsaturated fatty acids (PL-PUFAs), and the GPX4-glutathione axis can eliminate peroxidized PL-PUFA-OOH (left). The MALDI-TOF MS analysis clearly shows the ferroptosis-related metabolic network, which involves sphingolipid metabolism, the tricarboxylic acid (TCA) cycle, arachidonic acid (AA) metabolism, and tryptamine metabolism-related molecules (right)

Notch signals are involved in multiple cellular processes, including cell survival, differentiation, apoptosis, and regeneration (Siebel and Lendahl 2017). In addition, Notch signals are crucial for chondrogenesis and cartilage development (Hardingham et al. 2006). Recent studies have reported that Notch can promote the proliferation and inhibit the apoptosis of cells derived from degenerative discs (Long et al. 2019). The expression levels of Notch is also increased in the disc cells of patients with different Modic changes (Xiong et al. 2020). Our studies suggest that nucleus pulposus degeneration induced by heme iron is mediated by the Notch signaling pathway, which is a promising degeneration-related target on which to redirect LDH therapy. This mechanism is presented in Fig. 8.

## Conclusions

Our findings suggest that high levels of Hb and heme can be used to mark neovasculogenesis in herniated nucleus pulposus, which further promotes nucleus pulposus degeneration through heme iron-dependent cell death. These results are useful for in-depth study of degenerative pathology and will provide new ideas for conservative treatment of LDH patients, such as intervention in vasculogenesis.

## Supplementary Information

**Additional file 1: Fig. S1.** Heme degeneration catalyzed by HO-1. Heme is formed by iron and porphyrin. The heme iron in Hb is very stable because of the tight arrangement of molecule. But, the non-protein-bound heme is hydrophobic and can enter cell membranes easily. As a result, heme is degraded by HO-1 to yield free iron, which is able to enhance oxidative stress and induce ferroptosis in cells. **Fig. S2.** A representative H&E stain of nucleus pulposus with Pfirrmann’s grade 1 (lower) and 3–4 (upper). Scale bar = 20 μm. **Fig. S3.** MS spectra of oxidative biomarkers in herniated nucleus pulposus and nonherniated nucleus pulposus measured by the high-resolution mass spectrometry. The tissue samples were obtained from the patients 14 and 4 in Table 1. **Fig. S4.** MS spectra of oxidative biomarkers in HNPCs after treatment with 20 μg/mL heme, 20 μg/mL FAC, and 10 μg/mL erastin for 24 h using high-resolution MALDI-TOF MS.

**Additional file 2: Table S1.** Identification results of potential biomarkers in human lumbar discs. **Table S2.** Identification results of potential biomarkers in nucleus pulposus cells.

## Data Availability

All the data are included within the main article.

## Abbreviations

MALDI-TOF MS: Matrix-assisted laser desorption/ionization time-of-flight mass spectrometry
Hb: Hemoglobin
GPX4: Glutathione peroxidase 4
AA: Arachidonic acid
PUFA: Sphinganine, polyunsaturated fatty acid
TCA: Tricarboxylic acid cycle
LDH: Lumbar disc herniation
RBCs: Red blood cells
HNPCs: Human primary nucleus pulposus cells
DFO: Deferoxamine mesylate salt
FAC: Ferric ammonium citrate
PMSF: Phenylmethylsulfonyl fluoride
HMDB: Human metabolome database
HO-1: Heme oxygenase-1
OD: Optical density
PCA: Principal component analysis
ROC: Receiver operating curve (ROC)
ROS: Reactive oxygen species (ROS)
