# Supplementary material for: Increased hemoglobin and heme in MALDI-TOF MS analysis induce ferroptosis and promote degeneration of herniated human nucleus pulposus
Source: Mol Med. 2021 Sep 8;27:103. doi: 10.1186/s10020-021-00368-2 (PMC8425117; doi:10.1186/s10020-021-00368-2)
Supplement: Supplementary file 1 — Additional file 1: Fig. S1. Heme degeneration catalyzed by HO-1. Heme is formed by iron and porphyrin. The heme iron in Hb is very stable because of the tight arrangement of molecule. But, the non-protein-bound heme is hydrophobic and can enter cell membranes easily. As a result, heme is degraded by HO-1 to yield free iron, which is able to enhance oxidative stress and induce ferroptosis in cells. Fig. S2. A representative H&E stain of nucleus pulposus with Pfirrmann’s grade 1 (lower) and 3–4 (upper). Scale bar = 20 μm. Fig. S3. MS spectra of oxidative biomarkers in herniated nucleus pulposus and nonherniated nucleus pulposus measured by the high-resolution mass spectrometry. The tissue samples were obtained from the patients 14 and 4 in Table 1. Fig. S4. MS spectra of oxidative biomarkers in HNPCs after treatment with 20 μg/mL heme, 20 μg/mL FAC, and 10 μg/mL erastin for 24 h using high-resolution MALDI-TOF MS. [file 10020_2021_368_MOESM1_ESM.docx]

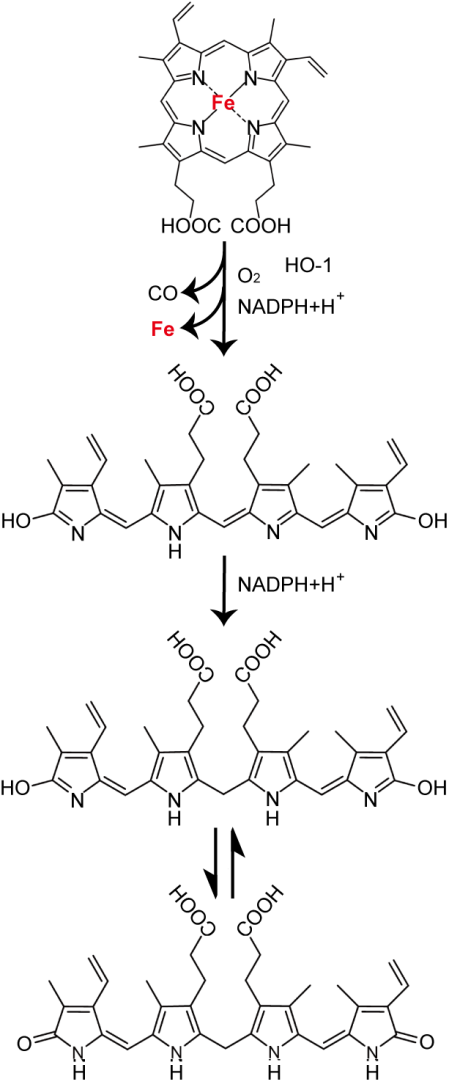


**Fig. S1.** Heme degeneration catalyzed by HO-1. Heme is formed by iron and porphyrin. The heme iron in Hb is very stable because of the tight arrangement of molecule. But, the non-protein-bound heme is hydrophobic and can enter cell membranes easily. As a result, heme is degraded by HO-1 to yield free iron, which is able to enhance oxidative stress and induce ferroptosis in cells.


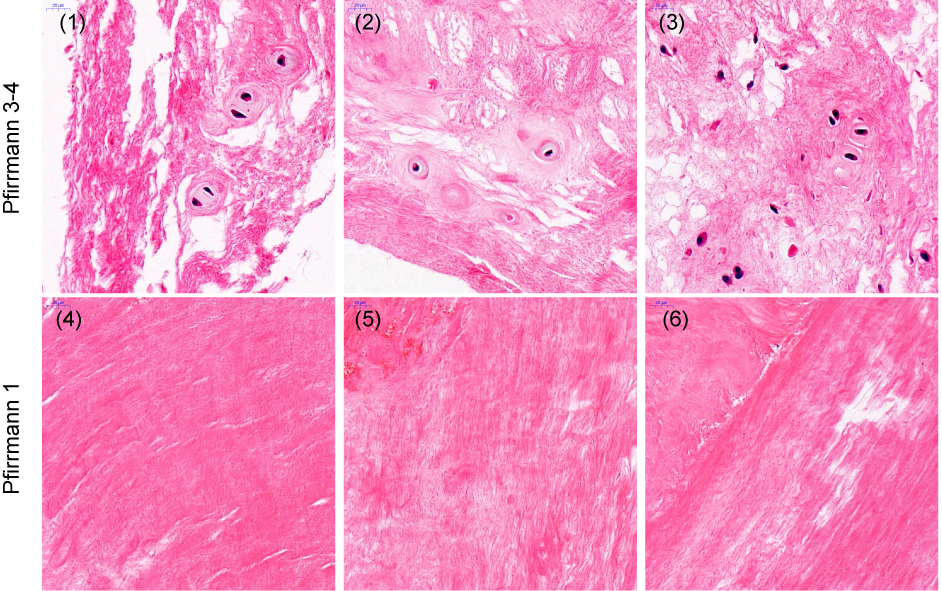


**Fig. S2.** A representative H&E stain of nucleus pulposus with Pfirrmann’s grade 1 (lower) and 3-4 (upper). Scale bar = 20 μm.


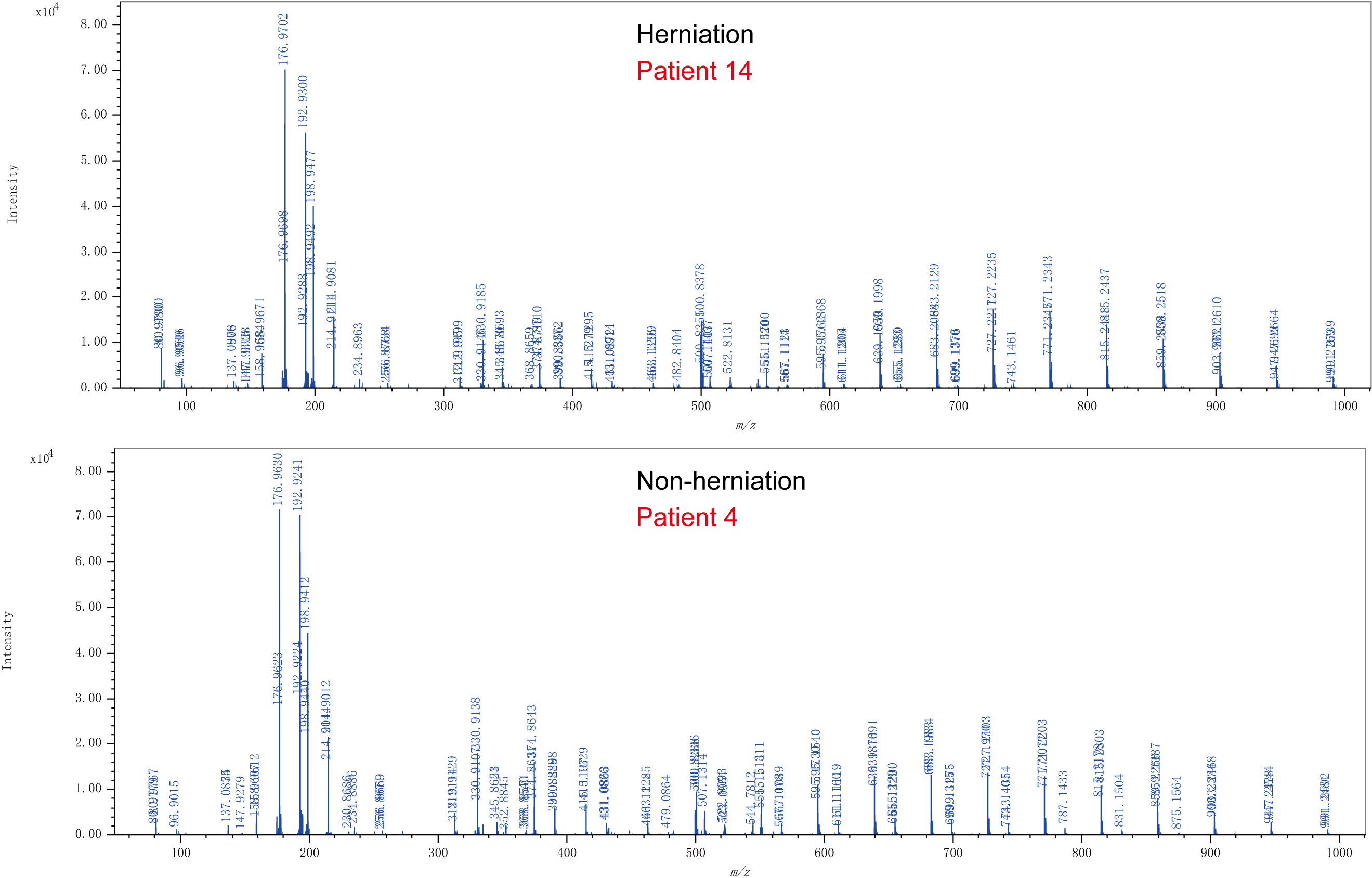


**Fig. S3.** MS spectra of oxidative biomarkers in herniated nucleus pulposus and nonherniated nucleus pulposus measured by the high-resolution mass spectrometry. The tissue samples were obtained from the patients 14 and 4 in Table 1.


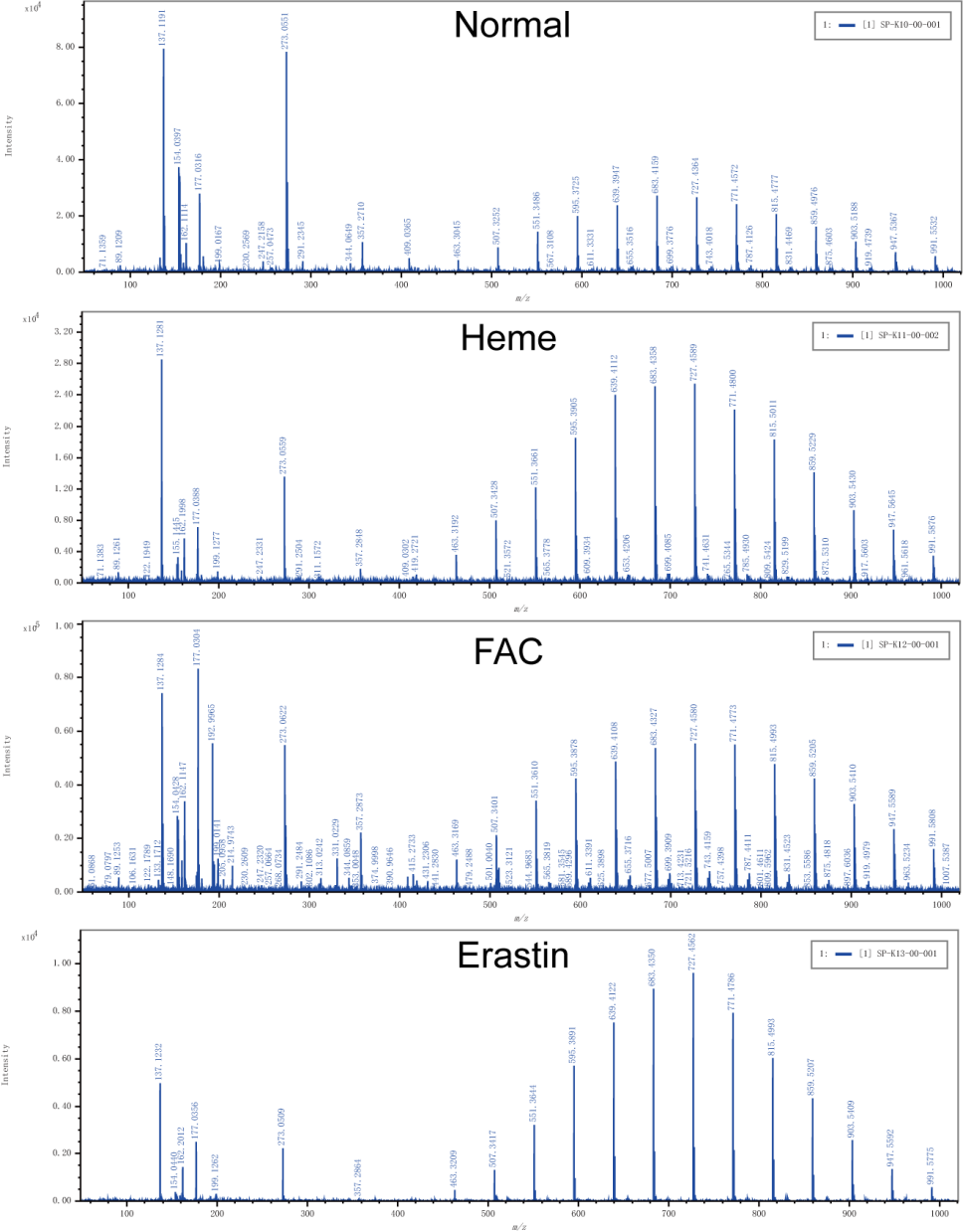


**Fig. S4.** MS spectra of oxidative biomarkers in HNPCs after treatment with 20 μg/mL heme, 20 μg/mL FAC, and 10 μg/mL erastin for 24 h using high-resolution MALDI-TOF MS.
