# Supplementary material for: Increased hemoglobin and heme in MALDI-TOF MS analysis induce ferroptosis and promote degeneration of herniated human nucleus pulposus
Source: Mol Med. 2021 Sep 8;27:103. doi: 10.1186/s10020-021-00368-2 (PMC8425117; doi:10.1186/s10020-021-00368-2)
Supplement: Supplementary file 2 — Additional file 2: Table S1. Identification results of potential biomarkers in human lumbar discs. Table S2. Identification results of potential biomarkers in nucleus pulposus cells. [file 10020_2021_368_MOESM2_ESM.pdf]

Table S1. Identification results of potential biomarkers in human lumbar discs.

No.	m/z	Formula	Mass Error	Biomarkers	Adducts	HMDB ID	Pathway	Relative signal intensity	
								Herniation	Normal
1	522.81	C26H52NO7P	0.47	LysoPC (18:1(9Z))	M + H	HMDB02815	Gly metabolism	5589	2155
2	612.12	C20H32N6O12S2	-0.04	Oxidized glutathione		HMDB03337	Glu metabolism	39286	12264
3	330.92	C10H17N3O6S	0.59	Glutathione	M + Na	HMDB00125	Glu metabolism	32185	14980
4	256.88	C8H20NO6P	-0.22	Glycerophosphocholine		HMDB00086	Gly metabolism	6832	2598
5	147.93	C5H6O5	0.91	α-Ketoglutarate	M + H	HMDB00208	TCA cycle	1509	1859
6	301.89	C18H39NO2	0.60	Sphinganine		HMDB00269	Sphinganine metabolism	7061	3761
7	305.06	C20H32O2	-0.18	Arachidonic acid	M + H	HMDB01043	AA metabolism	10735	0
8	327.86	C20H32O2	0.62	Arachidonic acid	M + Na	HMDB01043	AA metabolism	12775	3168
9	829.21	C42H79NO13	0.66	Lactosylceramide	M + Na	HMDB04866	Sphinganine metabolism	1830	0
10	159.06	C10H9NO	-0.01	Indoleacetaldehyde		HMDB01190	Tryptophan metabolism	9557	28650
11	352.88	C20H32O5	0.66	20-Hydroxy-leukotriene B4		HMDB01509	AA metabolism	2660	0
12	785.24	C44H82NO8P	0.14	18:2/18:1 phosphatidylcholine	M + H	HMDB08105	AA/Gly metabolism	2390	0
13	420.13	C24H47NO3	-0.23	Behenoylglycine	M + Na	HMDB13219	Sphinganine metabolism	1415	2353
14	330.24	C19H39NO3	-0.06	Dihydroceramide	M + H	HMDB06752	Sphinganine metabolism	32185	14980
15	415.12	C24H49NO4	-0.53	C6 Phytoceramide (t18:0/6:0)	M + H	HMDB12280	Sphinganine metabolism	20928	47016
16	131.94	C4H4O5	-0.06	Oxaloacetate		HMDB00223	TCA cycle	1136	0
17	159.9	C10H12N2	-0.14	Tryptamine		HMDB00303	Tryptophan metabolism	3167	42065
18	831.16	C23H38N7O17P3S	-0.97	Acetyl-CoA	M + Na	HMDB01206	TCA cycle PUFA metabolism	1214	3931

Table S2. Identification results of potential biomarkers in nucleus pulposus cells.

No.	*m/z*	Formula	Mass Error	Biomarkers	Adducts	HMDB ID	Pathway	Relative signal intensity			
								Normal	Heme	FAC	Erastin
1	522.31	C26H52NO7P	-0.04	LysoPC (18:1(9Z))	M + H	HMDB02815	Gly metabolism	3544	11500	28970	0
2	613.34	C20H32N6O12S2	0.19	Oxidized glutathione	M + H	HMDB03337	Glu metabolism	0	21024	53514	5664
3	330.01	C10H17N3O6S	-0.07	Glutathione	M + Na	HMDB00125	Glu metabolism	5678	3091	25595	19150
4	257.05	C8H20NO6P	-0.06	Glycerophosphocholine		HMDB00086	Gly metabolism	0	8662	22544	9164
5	147.10	C5H6O5	0.08	α-Ketoglutarate		HMDB00208	TCA cycle	0	1141	5055	0
6	302.10	C18H39NO2	-0.2	Sphinganine	M + H	HMDB00269	Sphinganine metabolism	0	17856	53868	59419
7	344.09	C20H32O3	0.85	(19S)-Hydroxy arachidonic acid	M + Na	HMDB11136	AA metabolism	0	1532	22245	4059
8	304.30	C20H32O2	0.06	Arachidonic acid		HMDB01043	AA metabolism	0	0	3252	0
9	829.47	C42H79NO13	0.91	Lactosylceramide	M + Na	HMDB04866	Sphinganine metabolism	0	2573	11302	2393
10	141.08	C2H8NO4P	0.06	O-phosphoethanolamine		HMDB00224	Sphinganine metabolism	0	0	10841	4496
11	375.26	C20H32O5	0.04	20-Hydroxy-leukotriene B4	M + Na	HMDB01509	AA metabolism	224	4354	21186	2548
12	784.46	C44H82NO8P	-0.12	18:2/18:1 phosphatidylcholine	M + H	HMDB08105	AA/Gly metabolism	0	0	3572	3237
13	331.02	C19H39NO3	0.72	Dihydroceramide	M + H	HMDB06752	Sphinganine metabolism	0	0	53514	0
14	132.08	C4H4O5	-0.07	Oxaloacetate		HMDB00223	TCA cycle	0	0	7499	2858
15	190.12	C10H7NO3	0.07	Kynurenate	M + H	HMDB00715	Tryptophan metabolism	59888	4409	22296	34416
16	159.22	C10H12N2	-0.88	Tryptamine		HMDB00303	Tryptophan metabolism	3544	11500	28970	0
17	831.45	C23H38N7O17P3S	0.32	Acetyl-CoA	M + Na	HMDB01206	TCA cycle PUFA metabolism	0	21024	53514	5664
18	853.57	C24H38N7O19P3S	0.45	Malonyl-CoA		HMDB01175	PUFA metabolism	0	3550	4072	10232
